# Supplementary material for: Deep learning framework for subject-independent emotion detection using wireless signals
Source: PLoS One. 2021 Feb 3;16(2):e0242946. doi: 10.1371/journal.pone.0242946 (PMC7857608; doi:10.1371/journal.pone.0242946)
Supplement: S1 File — (PDF) [file pone.0242946.s001.pdf]

Supplementary Information

Title:

Deep learning framework for subject-independent emotion detection using wireless signals

Author(s):

Ahsan Noor Khan, Achintha Avin Ihalage, Yihan Ma, Baiyang Liu, Yujie Liu and Yang Hao

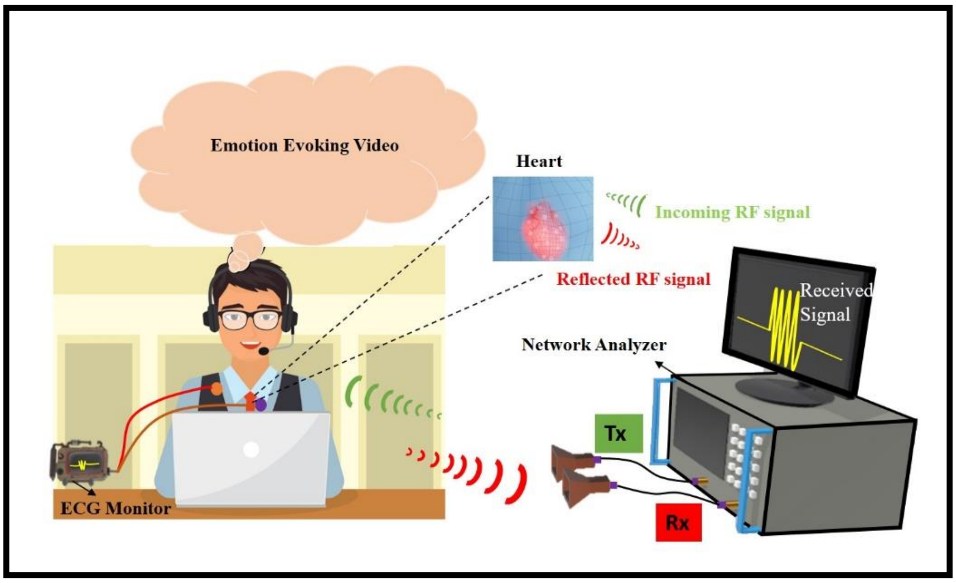

1. Deep Learning Analysis

1.1 Deep learning architecture selection

The selection of neural network architecture is arguably the most pivotal stage that determines the accuracy and performance of the model. The building blocks of the neural network (convolutional layers, LSTM cells, dense layers etc.) are usually selected by observing the potential inputs to the model. For example, 2D-convolutional layers generally perform best if the input includes images. The LSTM cells are preferred if the input is a time sequence or the time dependence of the data that needs to be learned. Likewise, if the inputs include a combination of such input types, the neural network is developed with a combination of corresponding building blocks. In this study, as mentioned in the main text, we have two distinct input types, namely, the time domain RF/ECG signal and frequency domain wavelet transformed image, thus the layers are selected accordingly.

Next task is to understand the number of layers, number of filters in each convolution layer and hyper-parameters required for optimal performance of neural network. This task is usually accomplished by intuition, trial and error methods. We have trained the neural network with different number of filters in each convolution layer and observed leave-one-out cross validation (LOOCV) classification accuracy in each case. The optimal hyper-parameter values (i.e. no of epochs, batch size, learning rate) were also investigated using grid search method (60, 50, 0.0001 respectively). Table S1. illustrates the training performance with different number of filters/layers.

Table S1. LOOCV accuracy with different number of filters/layers

**Kernels/feature maps in convolutional layer 1**	**Kernels/feature maps in convolutional layer 2**		**LOOCV accuracy (%)**
16	32		61.17
32	48		68.33
**32**	**64**		**71.67**
64	128		66.67
	**Kernels in convolutional layer 2**	**Kernels in additional convolutional layer 3**	
16	32	64	70.0

It should also be noted that performing cross validation with neural networks is slightly different than traditional machine learning. For example, the general idea of k-fold cross validation is to divide the database into k equal parts, training the model on k-1 splits, and later testing the model performance on the kth split for all k. However, the neural networks are trained for several epochs and the best model should be selected based on a validation set. Therefore, when performing LOOCV in this work, we selected one split from the k-1 splits as the validation data and test the model on the kth split.

1.2 Implementation, model parameters and training performance

The DNNs were implemented in python using keras-2.2.4 library with tensorflow-1.13 backend. We have also introduced L_2 regularization in each layer with a regularization parameter value of 10^{-4} to avoid overfitting. We make sure that we do not regularize too much, which would lead to under fitting, by setting the regularization parameter to a small value. After grid search optimization, the learning rate was set to 1 x 10^{-3}. Rectified linear unit (ReLU) was used as the activation function in hidden layers. Softmax activation is used at the output layer. The model was compiled with softmax cross entropy loss function. Figure S1 shows the training logs.

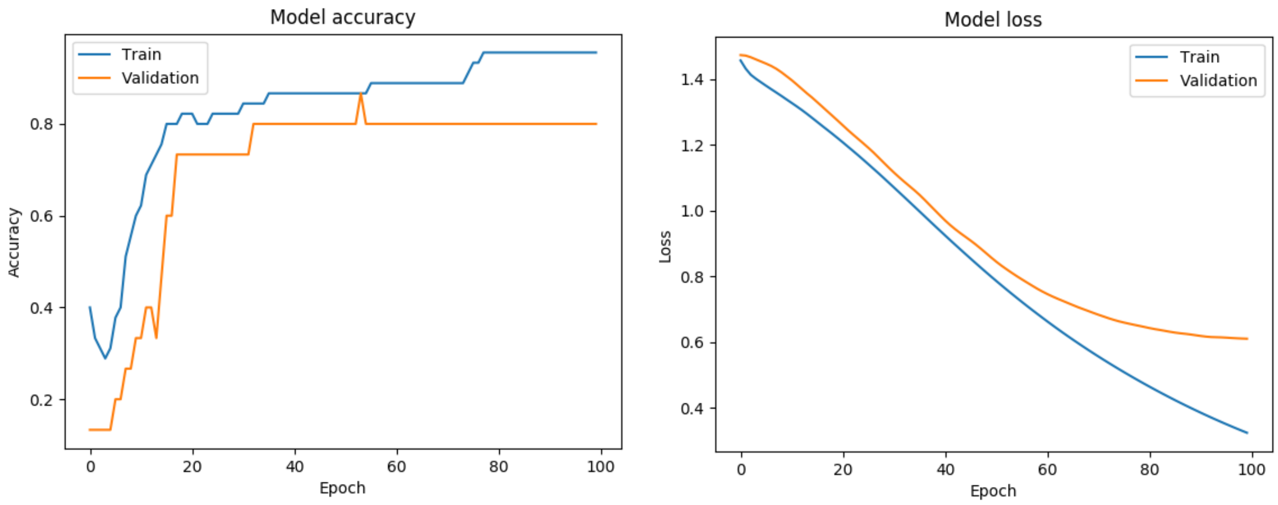

Figure S1: Model accuracy and loss deviation during training for RF emotion classification

2. Wavelet images and CNN feature maps

2.1 CW transformations of RF reflections relating to different emotions of a randomly selected subject

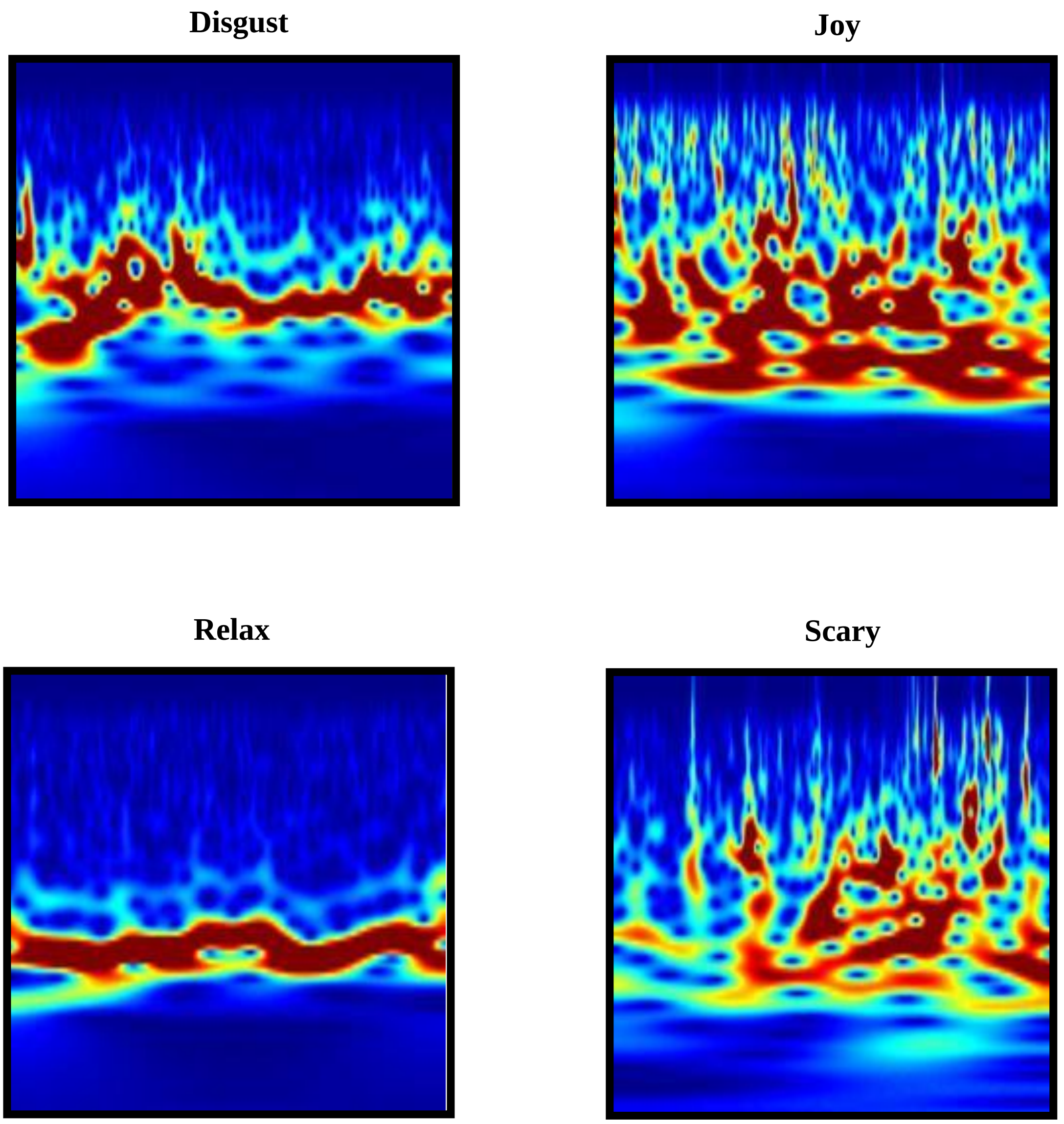

Figure S2: CW transformations of RF reflections relating to different emotions.

2.2 Visualizing the feature maps

First convolutional layer consists of 32 filters and thus 32 feature maps. Likewise, the second convolutional layer has 64 feature maps. Each filter extracts unique features from the input image. Figure S3 and Figure S4 depict the feature maps of two convolutional layers.

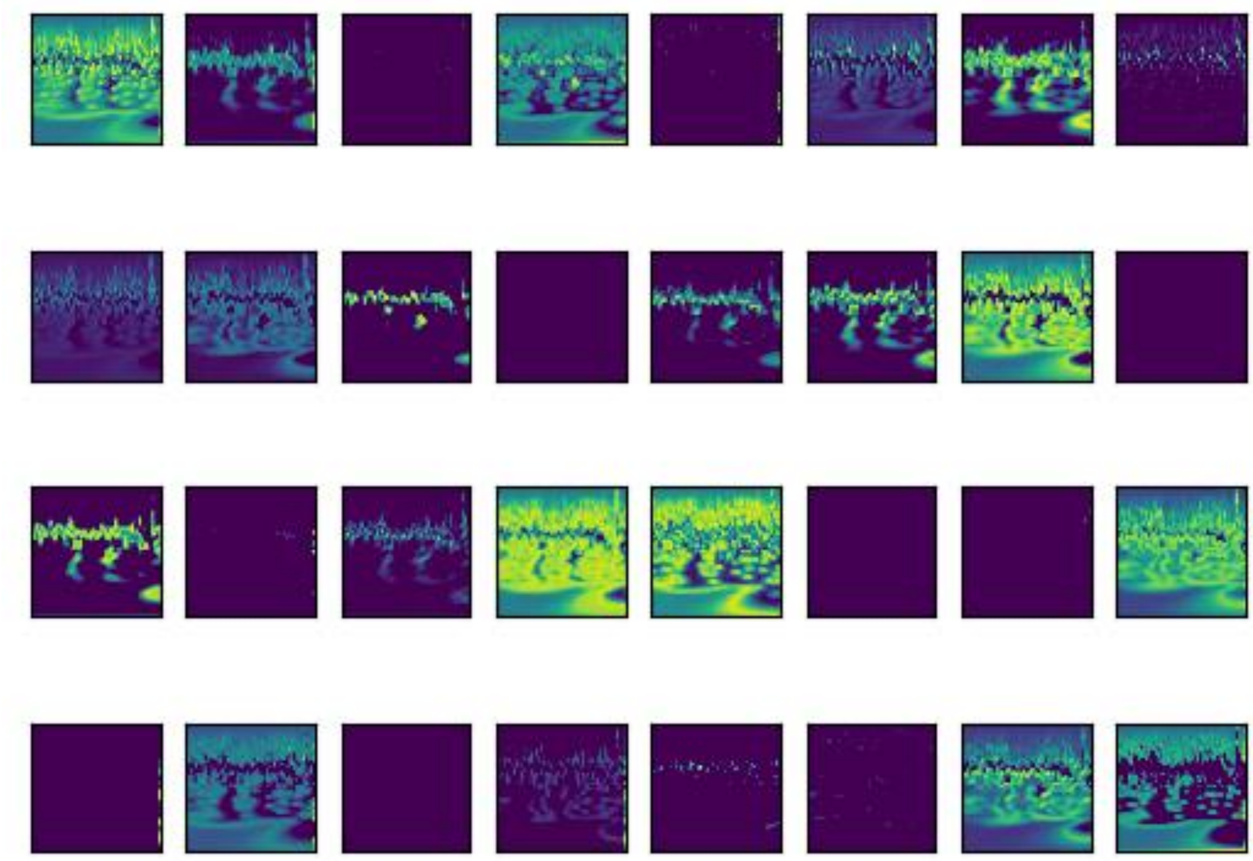

Figure S3: 32 feature maps in the first convolutional layer

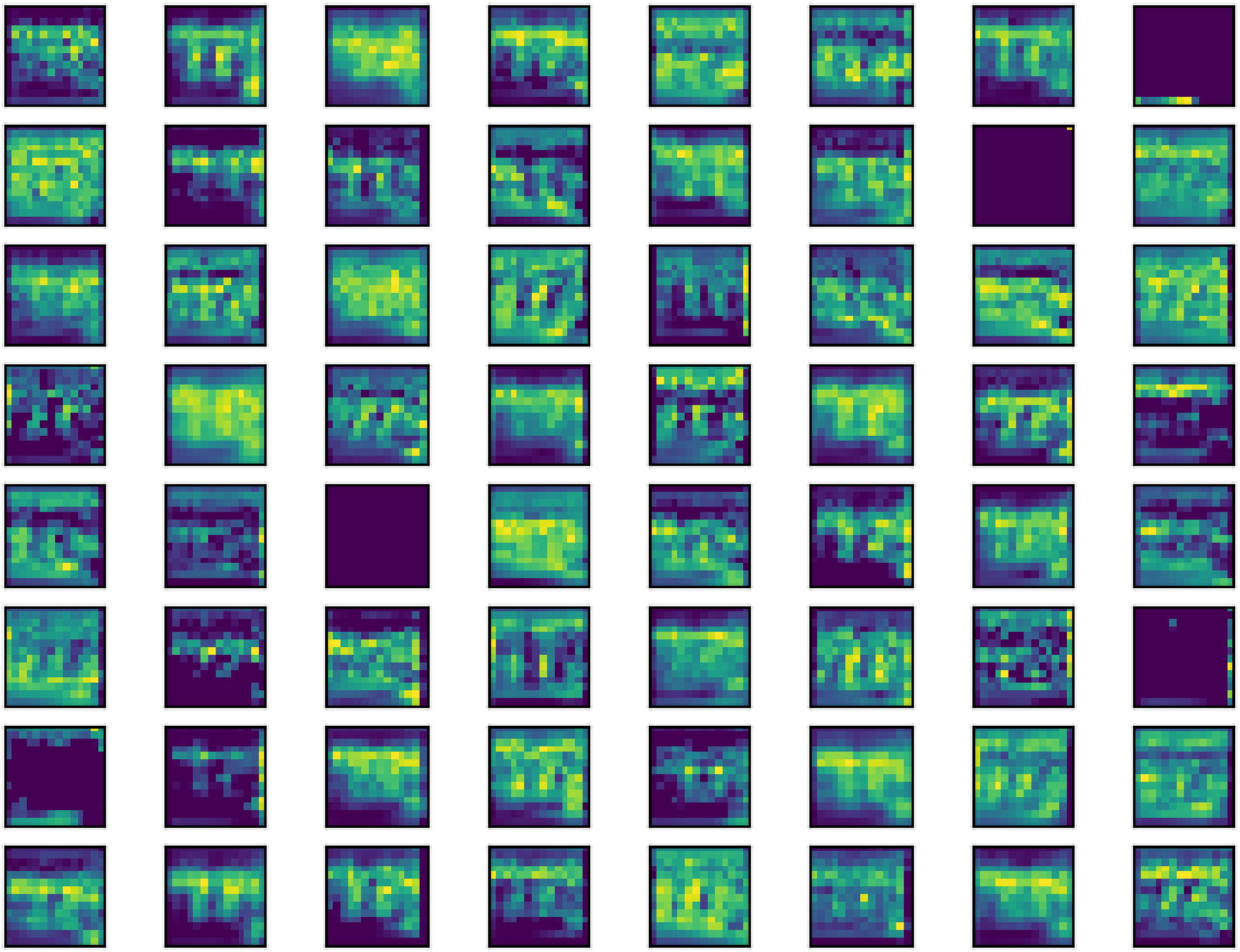

Figure S4: 64 feature maps in the second convolutional layer

3. Feature extraction process from RF reflected signal off the body

The process of feature extraction from reflected signals off the body is explained in this section. The normal breathing rate of human body is considered to be from 0.2 to 0.35 Hz, and the effect of body movements on the RF reflected signals is also considered in the low frequency range. Figure S5 (a) shows the raw reflected signals of one participant after normalization. During the data pre-processing, a band pass Butterworth filter with a frequency range from 0.1 – 8 Hz is implemented to reduce the random noise in the received RF signals, and to remove the baseline drift. Another benefit of using band pass Butterworth filter is to eliminate random and emotion unrelated movements of different participants by normalizing the signals in the range of [-1, 1]. Figure S5 (b) shows the filtered signals after normalization.

After pre-processing the RF reflections off the body, we have implemented the feature extraction process. In our work, we have extracted seven key parameters from the received RF signals, such as permutation entropy (PE), variance (var), skewness (ske), kurtosis (kur) and the power spectral density (PSD) in 0.15 – 2 Hz (PSD_1), 2 – 4 Hz (PSD_2) and 4 – 8 Hz (PSD_3) respectively [1]-[3]**.** Figure 5(c) illustrates the Fisher Space for the extracted parameters derived from different emotional states.

Although a plenty of entropy values were proposed to evaluate the expected values of the self-information, and the PE value has been used to tackle measurements. Considering the collected signal is $x = \{x|x = x_1, x_2, \dots, x_n\}$, then the **PE value** of order n is defined as

$$\mathrm{PE}(n) = -\sum p(\pi)\log p(\pi),$$

Where $p(\pi) = \frac{\#\{t|0 \le t \le T-n, (x_{t+1}, \dots, x_{t+n}) has\ type\ \pi\}}{T-n+1}$,

Variance, skewness and kurtosis are calculated by the following equations:

$$var = \sigma^2 = \sum_{n=1}^{L}\left(\bar{x}(n) - \frac{1}{L}\sum_{n=1}^{L}\bar{x}(n)\right)^2$$

$$ske = \frac{1}{L\sigma^3}\sum_{n=1}^{L}\left(\bar{x}(n) - \frac{1}{L}\sum_{n=1}^{L}\bar{x}(n)\right)^3$$

$$kur = \frac{1}{L\sigma^4}\sum_{n=1}^{L}\left(\bar{x}(n) - \frac{1}{L}\sum_{n=1}^{L}\bar{x}(n)\right)^4$$

Power spectral density is given as:

$$\mathrm{PS}D_i = \sum_{f_1}^{f_2}\frac{1}{N}\left|\sum_{n=1}^{N}\bar{x}(n)e^{-i2\pi(n-1)(k-1)/N}\right|^2$$

where the frequency band is determined by the range of (f_1, f_2)

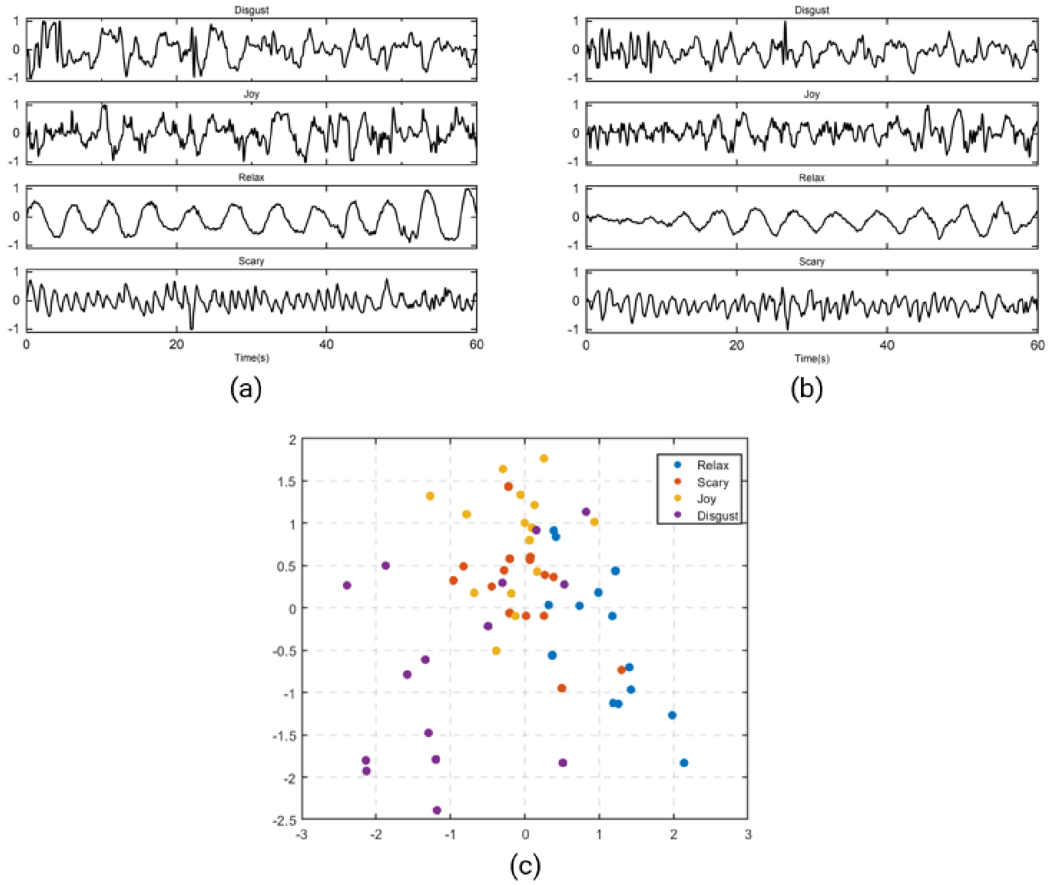

Figure S5: (a) Raw reflected signals after normalized; (b) reflected signals after filtered and normalized; (c) Fisher Space projection from extracted parameters

4. T-distributed Stochastic Neighbour Embedding (t-SNE) Algorithm

t-SNE is a nonlinear dimensionality reduction algorithm used for visualising high dimensional data in low-dimensional 2D or 3D space [4]. The t-SNE algorithm first calculates a similarity measure between pairs of instances in the high-dimensional space in such a way that similar instances have a high probability of being picked as the dimensions are reduced. Second, it calculates a similar probability measure over the points in the low-dimensional space, and minimized a cost function known as Kullback-Leibler (KL) divergence. The operation could be mathematically described as follows.

Given a set of N high dimensional instances x_1, x_2, .., x_N, t-SNE first computes probabilities p_{ij} that represent the similarity between the instances x_i and x_j as follows.

$$p_{j|i} = \frac{\exp(-\left\|x_i - x_j\right\|^2 / 2\sigma_i^2)}{\sum_{k\neq i} \exp(-\left\|x_i - x_k\right\|^2 / 2\sigma_i^2)}$$

$p_{j|i}$ represents the similarity of instance x_j to instance x_i; the probability that x_i would pick x_j as its neighbour if neighbours were picked in proportion to their probability density under a Gaussian centred at x_i.

t-SNE tries to learn a d-dimensional map $y_1, y_2, \ldots, y_N$ that reflects the similarities p_{ij} as well as possible. At this stage, it used a heavy-tailed Student t-distribution to measure similarities (q_{ij}) between low-dimensional points such that dissimilar objects are pushed far apart. q_{ij} is calculated as:

$$q_{ji} = \frac{(1 + ||y_i - y_j||^2)^{-1}}{\sum_{k \neq i}(1 + ||y_i - y_k||^2)^{-1}}$$

The locations of the point's y_i in the map are determined by minimizing the Kullback-Leibler divergence of the distribution Q from the distribution P, which is;

$$KL(P||Q) = \sum_{i \neq j} p_{ij} \log \frac{p_{ij}}{q_{ij}}$$

The minimization of KL divergence is performed using gradient descent. The optimized map is a low-dimensional representation of the high-dimensional inputs.

5. Self-assessment scaling rating on video clip

Self-assessment is an effective and reasonable approach to evaluate the emotional states from the subjective perception of participants. Scores range from 0 to 9 correspond with intensity of different emotional states range from low to high. The average assessments by all the participants in the study for each video clip in terms of four discrete emotional states are presented. The result shows desirable assessment scores for each item, and the major emotional state aroused by its corresponding video clip. However, each item also evokes other emotional states except the expected state, which also indicates it is hard to extract pure discrete emotions from the external affective stimuli [5].

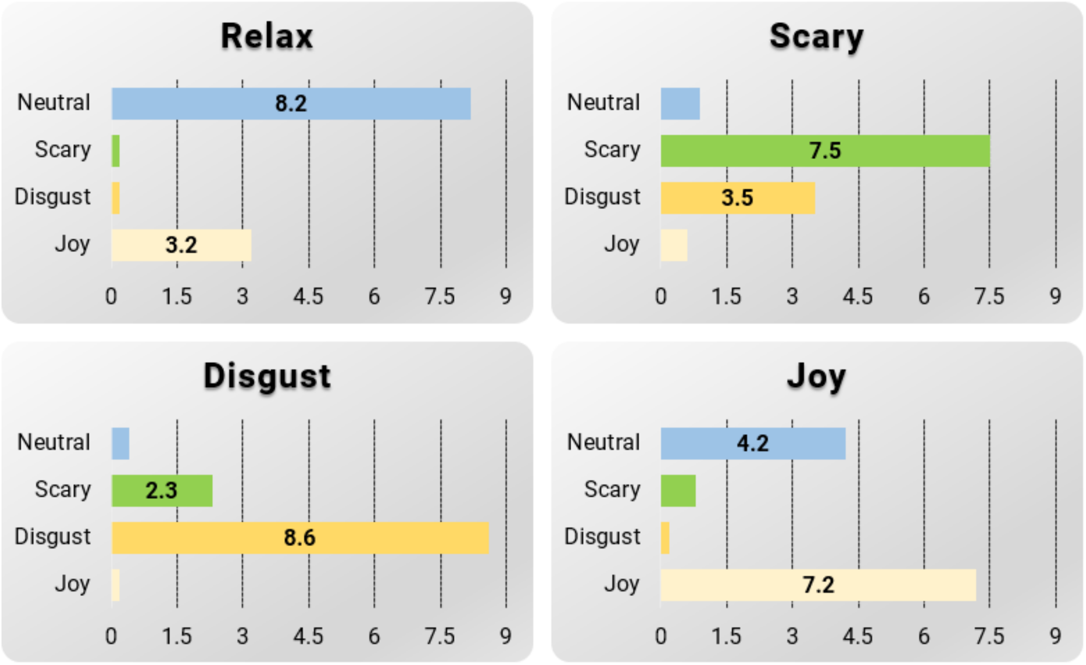

Figure S6: Average self-assessment scores from participants.

6. Validation of the deep learning model with DREAMER ECG database

The employability and generalizability of proposed deep learning architecture is investigated on the well-established DREAMER ECG database [6] for emotion recognition. The database contains the ECG recordings of 23 subjects for 9 emotions. In this validation section, we have selected four corresponding emotions (disgust, joy, relax, scary) similar to the present work. We have selected 83 IBI features from the ECG signals and employed mRmR algorithm to select 30 most significant features. Furthermore, the Wavelet Transformations of the ECG signals were also obtained. Likewise, the deep learning model was implemented with the 30 features and the Wavelet image as the inputs, followed by two convolutional-1D and two convolutional-2D layers respectively. The optimized hyper-parameters after the grid search method are as follows:

- No. of filters in first convolutional-1D layer: 32
- No. of filters in second convolutional-1D layer: 48
- No. of filters in first convolutional-2D layer: 16
- No. of filters in second convolutional-1D layer: 24
- Learning rate: 5 x 10^{-4}
- No. of epochs: 50
- Batch size: 40

We have obtained 68.48% LOOCV accuracy from the optimized DL model with 0.678, 0.685, 0.680 precision, recall and F1-score values respectively. Figure S7 shows the confusion matrix of the classification.

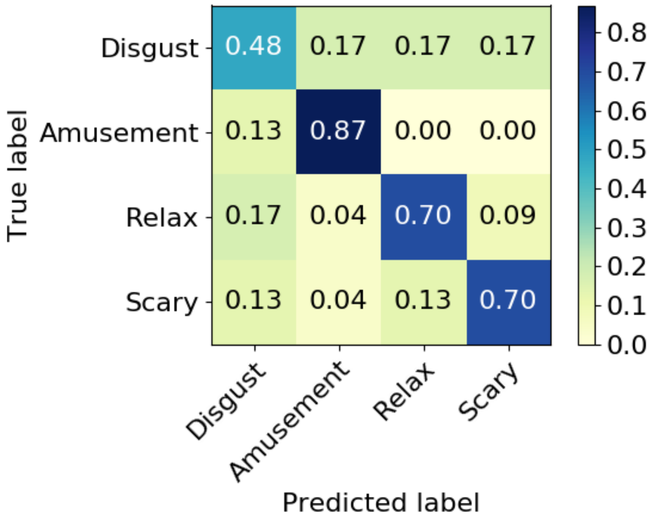

Figure S7: Confusion matrix of DREAMER ECG classification

7. Brief description about the emotion evoking videos

The brief description of videos is explained in Figure S8. We have searched and selected 4 different videos for this experiment, with movie clips which can stimulate four targeted emotion including Relax, Joy, Scary and Disgust. The videos were accessed and played online using YouTube platform**.**

Information on film clips and target emotions			
Title	Target Emotion	Video Length	Video Content
The World's Most Relaxing Film	Neutral	5 minutes	Natural landscape with gentle music
American's got Talent Funny	Joy	4 minutes	Funny talent show
Ju-On The Curse: Scariest Scenes	Scary	5 minutes	Japanese horror movie clips
Mangoworms, uncountable this time	Disgust	4 minutes	Squeeze worms from a dog body

Figure S8: Brief description about emotions evoking videos.

The experiment protocol can be briefly illustrated in Figure S9. Each participant was first explained about the experiment. After the end of each video (3-4 minutes), 2 minutes were given to the participant to record the intensity of emotions in the survey for assessment.

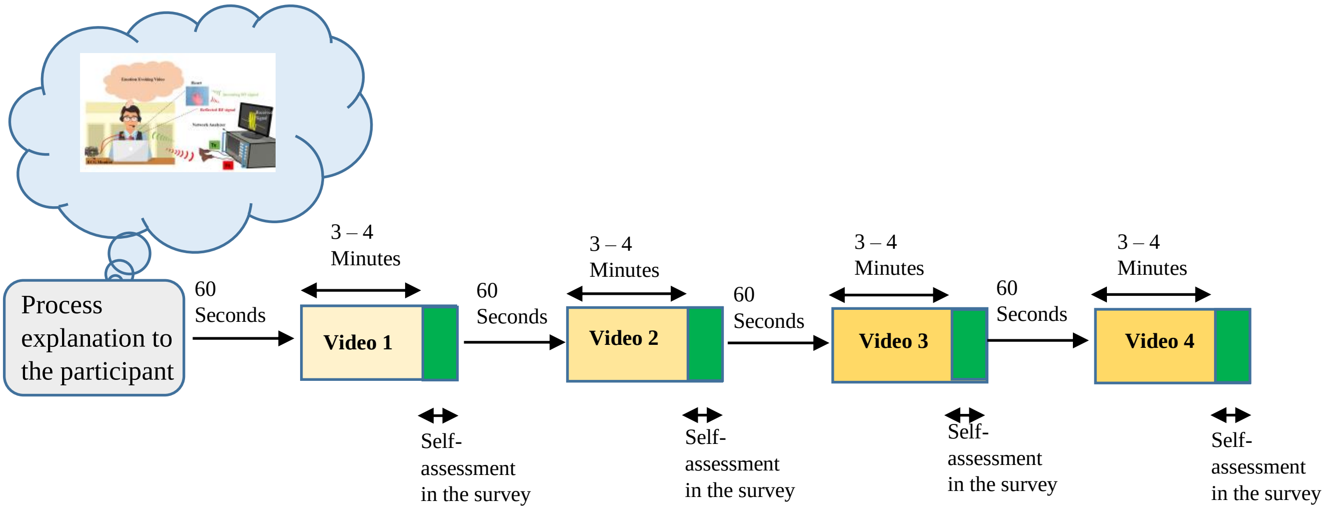

Figure S9: Brief description about the process of evoking emotions.

8. RF Measurement Setup:

8.1 Antenna Type

A pair of modified Vivaldi antennas was used in this study for the transmission and reception of RF signals in radar settings. The antennas were having reflection coefficient of less than -10 dB form 2.4 – 12 GHz. In this experiment we have used the frequency of 5.8 GHz. The dimensions of the antenna were 45 mm × 42 mm (L × W) as illustrated in Figure S10. The antennas were connected to the programmable network analyser.

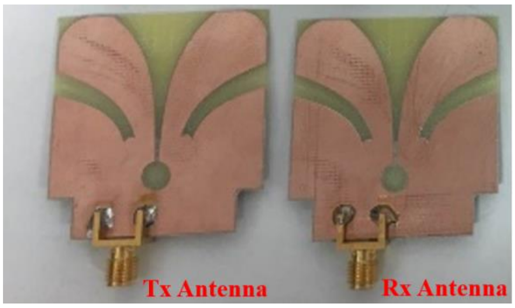

Figure S10: Vivaldi antennas for the transmission and reception of RF signals [7].

8.2 Measurement set-up with participating volunteers

We have recruited male volunteers for the convenience of measurements. Future work will be extended to a large scale trial in uncontrolled environment with a plan to a large scale trial in uncontrolled environment with a plan to recruit a large group of volunteers from more diverse communities considering gender equality.

The measurements set-up is shown in Fig. S11. It can be seen that the participant is sitting on the chair with ECG sensors mounted on the body. The transmitting and receiving antennas are targeted towards the chest area. All measurements were performed in the anechoic chamber. Each participant was asked to wear the headphones while watching the videos on the computer screen.

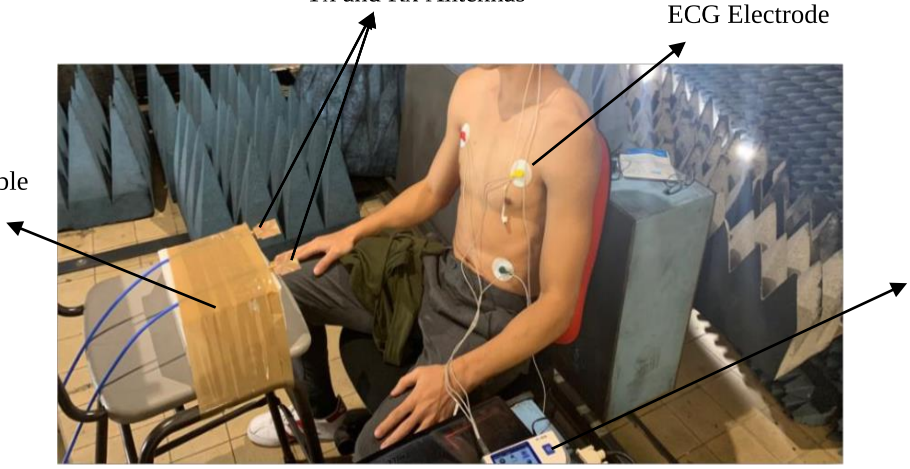

Figure S11: Measurement set-up in the anechoic chamber.

9. Emotion classification based on ECG signals

Electrocardiogram (ECG) signal is used to obtain electrical function of the heart, which also indicates strong association with emotional states. The ECG signal contains the cycle of P-waves, QRS-complex and T-waves, which can be separated into RR intervals, PR intervals, QT intervals, and so on [8]**.** While baseline wandering of ECG signals may sometimes exist during the experiment, which caused by variation of temperature, unexpected body movement, and deep respiration. Moreover, the effective frequency band is considering a range of 0.5 - 45 Hz. Therefore, in our experiment, after resampling raw ECG signals to 154 Hz, a 0.5 - 45 Hz band pass Butterworth filter is applied to implement signal filter and remove baseline drift.

After pre-processed the ECG signals, the next step is to extract features. Augsburg Biosignal Toolbox (AuBT) toolbox (a toolbox for analysing physiological signals written in Matlab) is introduced to automatic extraction of both time interval and morphological features from ECG signals [9]**.** The time interval features include, for example statistical parameters (mean, median, standard deviation, min, max values) from different segments of ECG signals, and the morphological features, such as the static rising and falling slopes of T-waves, rising and falling areas of P-waves, are extracted from the signals as well. 81 features obtained from AuBT toolbox are implemented for emotion classification.

The Support Vector Machine (SVM) machine learning algorithm is applied to classify emotional states. Based on the Leave-one-out Cross Validation (LOOCV) technique, the features extract from psychological signals achieve 68.3% classification accuracy. The acceptable classification results and the self-assessment scores from participants also indicate video clips evoke the participant's discrete emotional states successfully and can be regarded as the ground truth data in RF signal classification.

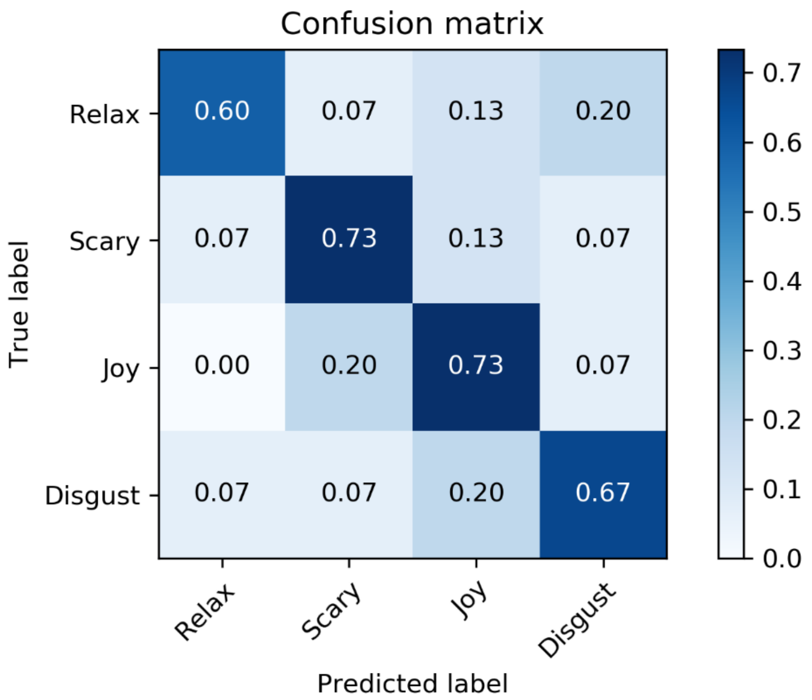

Figure S12: Classification accuracy on the features extract from psychological signals.

10. References

[1] C. Bandt and B. Pompe, "Permutation Entropy: A Natural Complexity Measure for Time Series," *Phys. Rev. Lett.*, 2002.

[2] T. N. Alotaiby, S. R. Alrshoud, S. A. Alshebeili, and L. M. Aljafar, "ECG-Based Subject Identification Using Statistical Features and Random Forest," *J. Sensors*, 2019.

[3] S. M. Alarcao and M. J. Fonseca, "Emotions Recognition Using EEG Signals: A Survey," *IEEE Trans. Affect. Comput.*, 2017.

[4] https://en.wikipedia.org/wiki/T-distributed_stochastic_neighbor_embedding

[5] C. A. Gabert-Quillen, E. E. Bartolini, B. T. Abravanel, and C. A. Sanislow, "Ratings for emotion film clips," *Behav. Res. Methods*, 2015.

[6] R. Kohavi, et al., Ijcai (Montreal, Canada, 1995), vol. 14, pp. 1137–1145.

[7] X. Zhuge and A. Yarovoy, "Design of low-profile antipodal Vivaldi antenna for ultra-wideband near-field imaging," in Proceedings of the Fourth European Conference on Antennas and Propagation, April 2010, pp. 1-5.

[8] F. Agrafioti, D. Hatzinakos, and A. K. Anderson, "ECG pattern analysis for emotion detection," *IEEE Trans. Affect. Comput.*, 2012.

[9] T. Lesiak, "Desmids of the Zbyszek peat-bog, Central Poland," *Fragm. Florist. Geobot.*, vol. 43, no. 1, pp. 65–76, 1998.
